# Development and validation of prognostic index based on immunogenic cell death-related genes with melanoma

**DOI:** 10.3389/fonc.2022.1011046

**Published:** 2022-11-07

**Authors:** Yajun Han, Qinqin Cai, Xiaolin Xie, Shilong Gao, Xiwen Fan

**Affiliations:** ^1^ Third Clinical Medical College, Xinjiang Medical University, Urumqi, China; ^2^ Department of Interventional Medicine, Affiliated Cancer Hospital, Xinjiang Medical University, Urumqi, China

**Keywords:** immunogenic cell death, melanoma, prognosis, tumor microenvionment, immunotherapy

## Abstract

Although immune checkpoint inhibitors have improved the overall survival rate of skin cutaneous melanoma (SKCM) patients, there is a wide variation and low response rate to these treatments in clinical immunotherapy for melanoma patients. These problems can be addressed through the induction of immunogenic cell death (ICD).We constructed an ICD-based prognostic model to predict the prognosis of SKCM patients and the efficacy of immunotherapy. Information on melanoma and normal samples obtained by TCGA and GTEx was stratified by ICD-related genes. The samples were divided into two subtypes according to high and low expression of ICD using an unsupervised clustering method (K-means). Patients with ICD-high subtype showed longer overall survival. We found that the ICD-related differential genes were associated with several cell death and immune-related pathways through GO, KEGG and GSEA. Immunoscore and tumor purity of ICD-associated genes was calculated using ESTIMATE, and ICD-high subtypes had higher immunoscore and lower tumor purity than ICD-low subtypes. Seven ICD-associated genes were obtained by one-way Cox regression and Lasso regression of ICD genes. Risk models were constructed to classify melanoma patients into high- risk and low-risk groups. The expression of ICD-related pivotal genes was lower in the high-risk group than in the low-risk group, and the survival time was significantly higher in the low-risk group than in the high-risk group. We then found that ICD risk characteristics had predictive value for the clinical efficacy of immunotherapy, with higher ICD risk scores in the immunotherapy non-responsive group. Combined with clinicopathological factors, a nomogram was established. the ROC and calibration curves assessed the ability of the nomogram to predict prognosis. We developed a new classification system for SKCM based on the characteristics of ICDs. This stratification has important clinical implications for estimating the prognosis and immunotherapy of SKCM patients.

## Introduction

Skin cutaneous melanoma (SKCM) is one of the most aggressive malignancies, characterized by insidious pathogenesis, metastasis and poor response to treatment, and accounts for 80% of all deaths from skin tumors (1, 2). It is a highly immunogenic cancer (3). In recent years, several studies have made progress in understanding the cellular and molecular mechanisms involved in tumorigenesis, metastasis and immune escape and have introduced a variety of new immunotherapies (4, 5). With the rapid development of Immune Checkpoint Inhibitors (ICIs), antibodies against programmed cell death protein 1(PD-1), Programmed cell death-ligand 1(PD-L1)and cytotoxic T-lymphocyte-associated protein 4(CTLA-4) have been approved one after another for the treatment of melanoma (4, 6). The objective response rate of these inhibitors can reach 30-40% (7, 8). Although ICIs offer new hope to melanoma patients, some patients have low response rates to immunotherapy due to the heterogeneity of the tumor and have serious adverse effects (9). Many studies have shown that the induction of immunogenic death (ICD) can significantly enhance the anti-tumor immune response.

ICD can promote immune stimulation in patients by enhancing the antigenicity of tumor cells (10). Immunotherapy activates or enhances the immune system to attack tumor cells, inducing specific anti-tumor immunity and prolonged immunogenic memory responses. ICD inducer-treated tumor cells stimulate the exposure of tumor-associated antigens, known as damage-associated molecular patterns (DAMP) (11), including calreticulin. DAMP promotes dendritic cells maturation, activation of cytotoxic T lymphocytes and secretion of a variety of cytokines (interferon-I) (12). ICDs enable anti-tumor T cells and secretion of multiple cytokines to alter the tumor microenvironment, thereby enhancing the response to immunotherapy (13). The results of this study are summarized below. In many previous studies, ICD inducers increased immunotherapeutic sensitivity (14–16). However, there is little research on using ICD inducers in preclinical models.

Therefore, to make current immunotherapy more precise and effective, it is necessary to identify new therapeutic targets and approaches to classify melanoma patients accurately. Our study developed risk models for ICD-related genes to predict prognosis and response to immunotherapy in SKCM patients and can be used as prognostic biomarkers.

## Materials and methods

### Datasets

**Figure 1:**
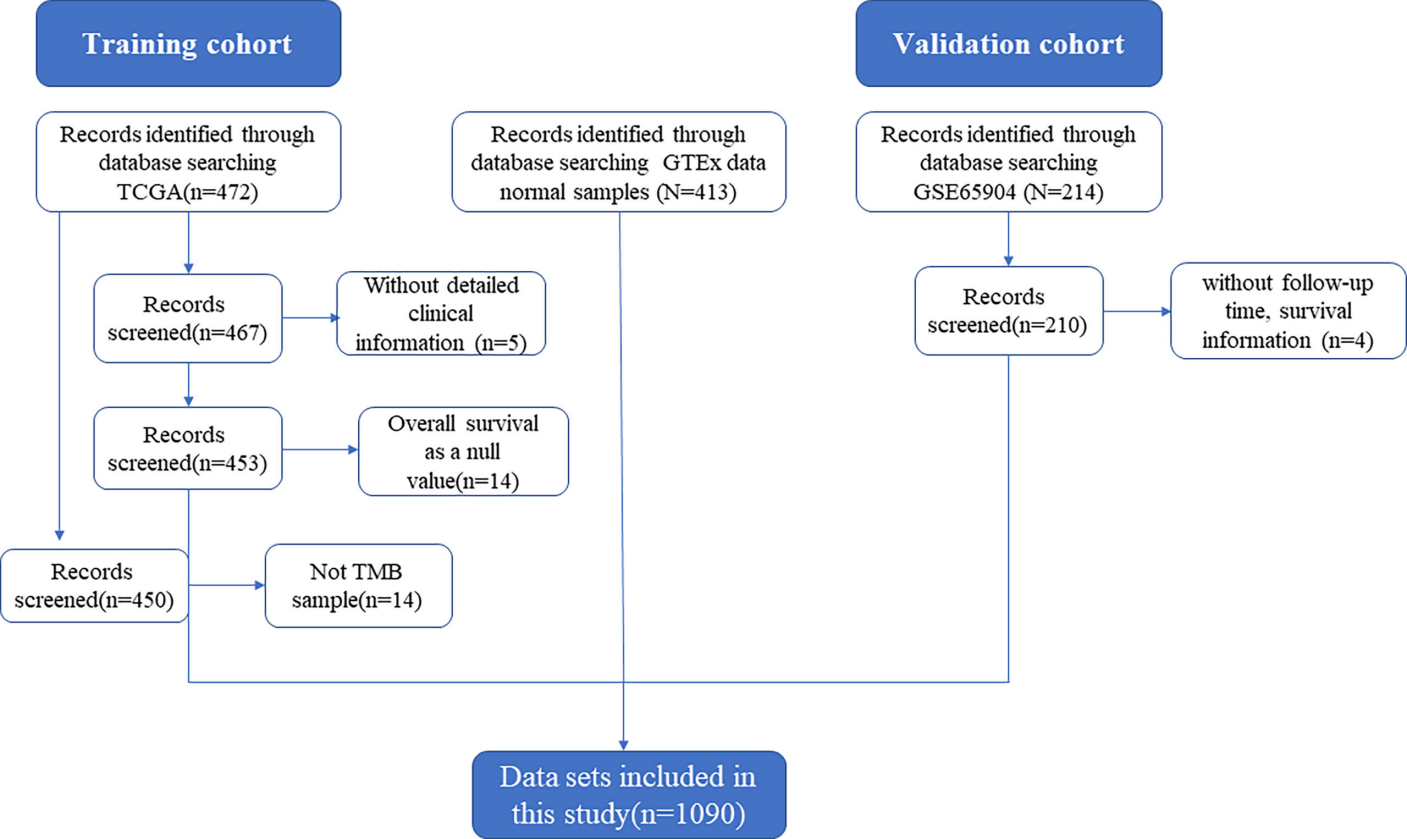
Flow-diagram of the datasets selection process.

The data filtering process is shown in **Figure 1**. The training set data used in this study consisted of 880 samples, including 413 normal samples and 467 SKCM samples. RNA sequencing data and associated clinical information were downloaded from the Cancer Genome Atlas data portal (TCGA, https://portal.gdc.cancer.gov/) and the GTEx data portal (https://www.gtexportal.org/home/datasets/). A total of 472 SKCM samples were downloaded from the TCGA database, and 467 datasets were included in the training group after excluding 5 datasets without follow-up time, survival information and detailed clinical information. Also, 17 samples without tumor mutation burden (TMB) information were excluded. A total of 214 SKCM samples from dataset GSE65904 were downloaded from the Gene Expression Omnibus (GEO) database, normalised and data from 4 patients without clinical outcomes were excluded, and 210 data were included in the validation set.

The study ultimately included 677 patients with SKCM, including 413 (61.00%) males and 264 (39.00%) females, with an age at initial pathological diagnosis between 15 and 91 years, with no statistically significant differences between the two groups for the characteristic variables (p > 0.05, **Table 1**).

**Table 1:** Data source and clinicopathologic characteristics of patients.

Characteristics	Training cohort TCGA (N=467)	Validation cohort GSE65904 (N=210)	Total (N=677)	*p*
Age				
Mean ± SD	58.17 ± 15.76	62.35 ± 14.40	59.48 ± 15.45	
Median[min-max]	58.00[15.00,90.00]	64.00[22.00,91.00]	61.00[15.00,91.00]	
Sex				0.54
Female	178 (26.29%)	86 (12.70%)	264 (39.00%)	
Male	289 (42.69%)	124 (18.32%)	413 (61.00%)	
OS				
Mean ± SD	1805.00 ± 1938.39	981.40 ± 1222.82	1549.53 ± 1788.24	
Median	1089	534	839	
Event				0.71
0	249 (36.78%)	108 (15.95%)	357 (52.73%)	
1	218 (32.20%)	102 (15.07%)	320 (47.27%)	

0, alive; 1, death; OS, Overall survival.

### Consensus clustering of genes immunogenic cell death

Cluster analysis using the R package ConsensionClusterPlus (17). Subsequently, we used a pam cluster approach with 1-pearson correlation distances to partition the ICD-associated genes. This process was repeated 1000 times for 80% of the samples to ensure the stability of the results. The optimal number of clusters was determined using an empirical cumulative distribution function plot.

### Identification of differentially expressed genes

Samples of 2 distinct ICD group were analyzed by the empirical Bayesian approach of the R package limma (18) (version 3.52.2) to identify DEGs between different ICD group, and adjusted p value < 0.05 and | fold change| > 1 were considered as differential genes.

### Functional enrichment analysis

For gene set functional enrichment analysis,we utilized the R package clusterProfiler (19) (version 4.4.4) to enrichment analysis, and we used the enrich GO function of the R package org.Hs.eg.db (version 3.15.0), setting the minimum gene set to 10, the maximum gene set to 500, pvalueCutoff = 0.05, pAdjustMethod = “BH”. Adjusted p value < 0.05 were considered statistically significant to obtain gene set enrichment results. Using the enrichKEGG function, setting the minimum gene set to 10 and the maximum gene set to 500, pvalueCutoff = 0.05, pAdjustMethod = “BH”. Adjusted p value < 0.05 were considered statistically significant and gene set enrichment results were obtained.

### Gene set enrichment analysis

For the Gene set enrichment analysis (GSEA), we downloaded two subsets, h.all.v7.5.1.entrez.gmt and c7.immunesigdb.v7.5.1.entrez.gmt, from the Molecular Signatures Database, and then enriched the two gene sets using differential genes to assess relevant pathways and molecular mechanisms. Based on gene expression profiles and phenotype groupings, set the minimum gene set to 10, the maximum gene set to 500, pvalueCutoff = 0.05, and pAdjustMethod = “BH”. Adjusted p value < 0.05 and were considered statistically significant to obtain gene set enrichment results.

### Characterization of immune landscape between two ICD subgroups

To characterize the immunological properties of the two subgroups of SKCM, the R package estimate (version 1.0.13 (20). https://R-Forge.R-project.org/projects/estimate/.) StromalScore, ImmuneScore, ESTIMATEScore and tumor purity between the two subgroups.

Using the R script (version 1.03) and Leukocyte signature matrix (LM22) (21) provided by CiberSort (https://cibersortx.stanford.edu/), the analysis was repeated 1000 times to determine the relative percentages of the 22 immune cell types. We then compared the relative percentages of the 22 immune cell types between the two ICD subgroups.

### Prediction of response to immunotherapy

Immunotherapy response was determined using tumour immune dysfunction and rejection (TIDE) analysis (22). TIDE (http://tide.dfci.harvard.edu/) is an analysis technique that uses two major mechanisms of tumour immune evasion (T cell dysfunction and T cell infiltration suppression in tumours with low CTL levels) to predict immunotherapy response.

### Somatic mutation analysis

Somatic mutation data of the SKCM samples were obtained from TCGA GDC Data Portal in “maf” format. Waterfall plots were then performed using the R package mafTools (23).

### Survival analysis

All SKCM patients were divided into two groups by the median risk score.Kaplan-Meier (KM) analysis was conducted for comparison of the overall survival (OS) between low and high subtype ICD and the low and high ICD risk cohort utilizing the R software packages survminer and survival (version 3.3-1).

### Construction of the ICD-related risk signature

Patient survival time, survival status, and gene expression data were integrated using the R package glmnet (24).

Prospective prognostic indicators were identified by including 23 ICD-related genes in univariate Cox analyses (25). LASSO cox regression analysis was then performed to calculate the exact coefficient values for each identified association. LASSO is a commonly used regression analysis method that combines variable selection and regularization to improve the predictive performance and interpretability of the resulting statistical model. Multivariate Cox analysis was used to identify independent risk factors for SKCM.

We then constructed a nomogram using the multivariate cox approach and assessed the prognostic significance of these features in 453 samples. The overall C-index of the model was: 0.706833299737956,95% CI (0.669570706055558-0.744095893420355), p value =1.4488773564683e^-27^. The discriminative power of the nomogram was assessed by ROC curves. Calibration curves were plotted to investigate the agreement between the actual operating system and the operating system predicted by the nomogram.

### Statistical analysis

Categorical variables were compared using Pearson’s chi-square test. To compare the OS of patients between subgroups, we used the Kaplan-Meier method. The risk model was constructed using univariate Cox and LASSO regression analysis. To assess the independent prognostic value of the risk model, we used univariate and multivariate Cox regression models. When comparing immune cell infiltration between the two groups, the Kruskal-Wallis rank sum test was used. A two-tailed p-value <0.05 was considered statistically significant. All statistical analyses were done using R software (v4.2.1).

## Results

### Consensus clustering identified two ICD-associated subtypes

**Figure 2:**
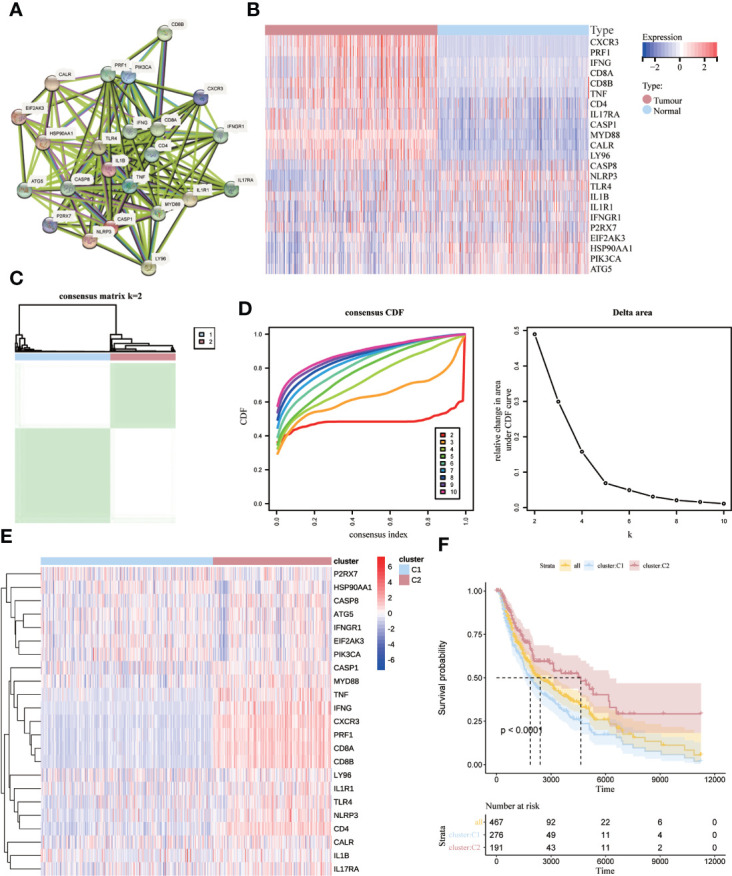
Identification of ICD-associated subtypes by consensus clustering. **(A)** Protein interactions between ICD-associated genes; **(B)** heat map showing the expression profiles of 23 ICD genes in normal and SKCM samples from the TCGA database; **(C)** heat map depicting the consensus clustering solution (k = 2) for 23 genes in 467 SKCM samples; **(D)** δ-area curves of the shared clusters representing the cumulative distribution function from k = 2 to 10 (CDF) curves under the relative change in the area; **(E)** Heat map of the expression of 23 ICD-related genes in different subtypes. Red represents high expression, and blue represents low expression; **(F)** Kaplan-Meier curves for ICD high subtype and ICD low subtype OS. p < 0.0001).

Based on Abhishek et al. (26) previously summarized the extensive literature identifying genes associated with ICD (TNF, CXCR3, P2RX7, CASP1, NLRP3, IL1B, LY96, CD4, CD8A, CD8B, PRF1, IFNG, IL17RA, HSP90AA1, EIF2AK3, PIK3CA, CASP8, ATG5 IL1R1, MYD88, IFNGR1, CALR, TLR4), we performed protein-protein interaction (PPI) network analysis using the STRING database to suggests a link between these ICD-associated gene nodes (**Figure 2A**). To confirm the expression of these ICD-related genes in melanoma, we compared these 23 ICD-related genes that differed between melanoma and normal samples, with a total of eight genes significantly different (PRF1, LY96, ILR1, HSP90AA1, CD8A, CD4, CALR, CXCR3), as shown in **Figure 2B**. Next, we used consensus clustering to identify the ICD-related clusters of SKCM. After k-means clustering, SKCM patients were divided into two clusters with different expression patterns of the ICD gene (**Figure 2C**). The number k of clusters was selected from 2 to 10 (**Figure 2D**).Overall, C2 showed high expression levels of ICD-associated genes, indicating a high ICD subtype. In contrast, the relevant genes showed low expression levels in the C1 cluster (**Figure 2E**). Therefore, we defined the C1 cluster as the ICD-low subtype and the C2 cluster as the ICD-high subtype. Furthermore, survival analysis showed that the ICD-high subtype was associated with better overall survival (OS), while the ICD-low subtype was associated with poorer OS (**Figure 2F**).

### Identification of differentially expressed genes and signaling pathways

**Figure 3:**
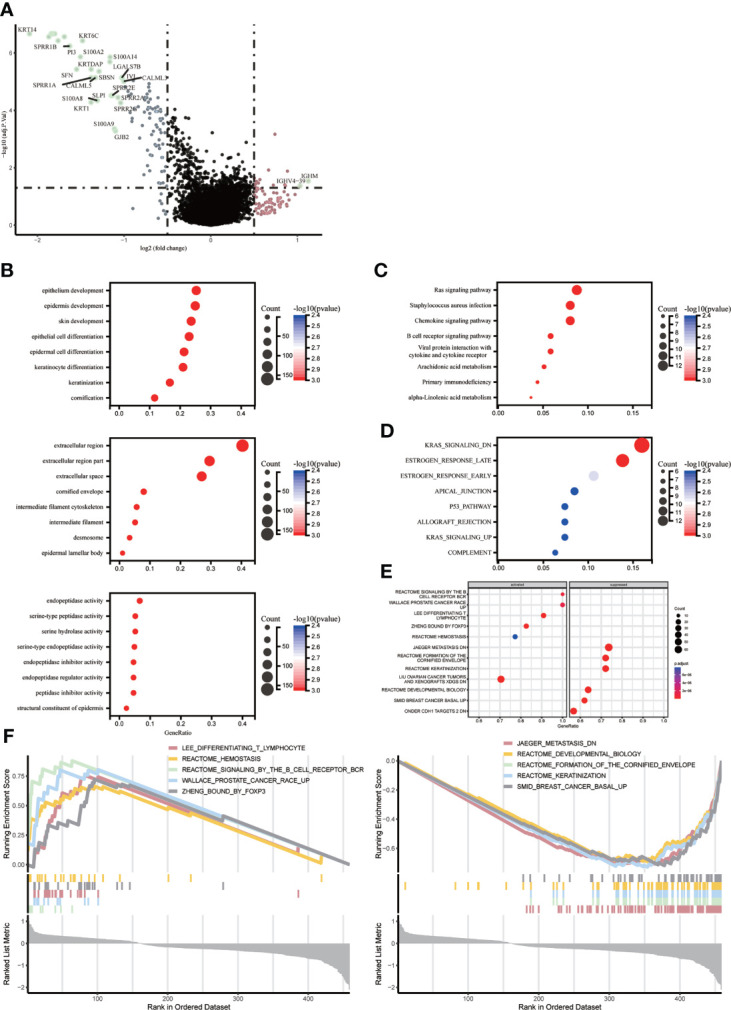
Identify differentially expressed genes (DEGs) and potential signaling pathways in different subtypes. **(A)** Volcano plot showing the quantitative distribution of DEGs between ICD-high and ICD-low subtypes in the TCGA cohortlog2-fold change in threshold > 1 and p < 0.05 **(B–E)** Bubble plots showing enrichment analysis of KEGG and GO signaling pathways. The size of the dots represents gene counts, and the color of the dots represents -log10(p adjust-value); **(F)** GSEA analysis identifies potential signaling pathways between ICD high and ICD low subtypes.

We identified key genes and signaling pathways in both subtypes to understand the molecular mechanisms associated with prognosis. Melanoma patients were divided into two subgroups, ICD-high and ICD-low, based on ICD gene expression. A total of 111 genes were significantly differentially expressed between ICD-high and ICD-low(p<0.05). There were 30 differential genes with | fold change|>1. Of these, 28 genes were down-regulated such as (KRT14, KRT17, S100A7, KRT16, KRT6A, KRT6B, KRT6C, KRT5, SPRR1B, PI3, S100A2, S100A14, SFN, KRTDAP, SBSN, IVL, SPRR1A, LGALS7B, CALML5, CALML3, SPRR2E, SLPI, SPRR2A, S100A8, KRT1, SPRR2G, S100A9, GJB2), and 2 genes were upregulated (IGHM, IGHV4-39) (**Figure 3A**). The main signaling pathways involved in melanoma are a number of cell death and immune response related pathways with varying levels of gene enrichment, such as the RAS signaling pathway, KRAS-related pathway, chemokine signaling pathway, B-cell receptor signaling pathway, primary immunodeficiency, interaction of viral proteins with cytokines and their receptors, T-cell receptor complex, and estrogen response (**Figures 3B–E**).

ICD-related genes are highly expressed and enriched for the function of LEE_DIFFERENTIATING_T_LYMPHOCYTE、PUJANA_ATM_PCC_NETWORK、REACTOME_SIGNALING_BY_THE_B_CELL_RECEPTOR_BCR、ZHENG_BOUND_BY_FOXP3, low expression enrichment function for JAEGER_METASTASIS_DN、REACTOME_DEVELOPMENTAL_BIOLOGY、REACTOME_FORMATION_OF_THE_CORNIFIED_ENVELOPE、SMID_BREAST_CANCER_BASAL_UP.The high expression group was shown to be mainly associated with the stage of T-lymphocyte differentiation, mutation of the HMMR locus, signalling of the B-cell receptor, and regulation of CD4+ T-cell subsets by the transcription factor Foxp3, while the low expression group was mainly associated with the molecular mechanisms of progression and metastasis of malignant melanoma (**Figure 3F**).

### Somatic cell mutations and the tumor microenvironment

**Figure 4:**
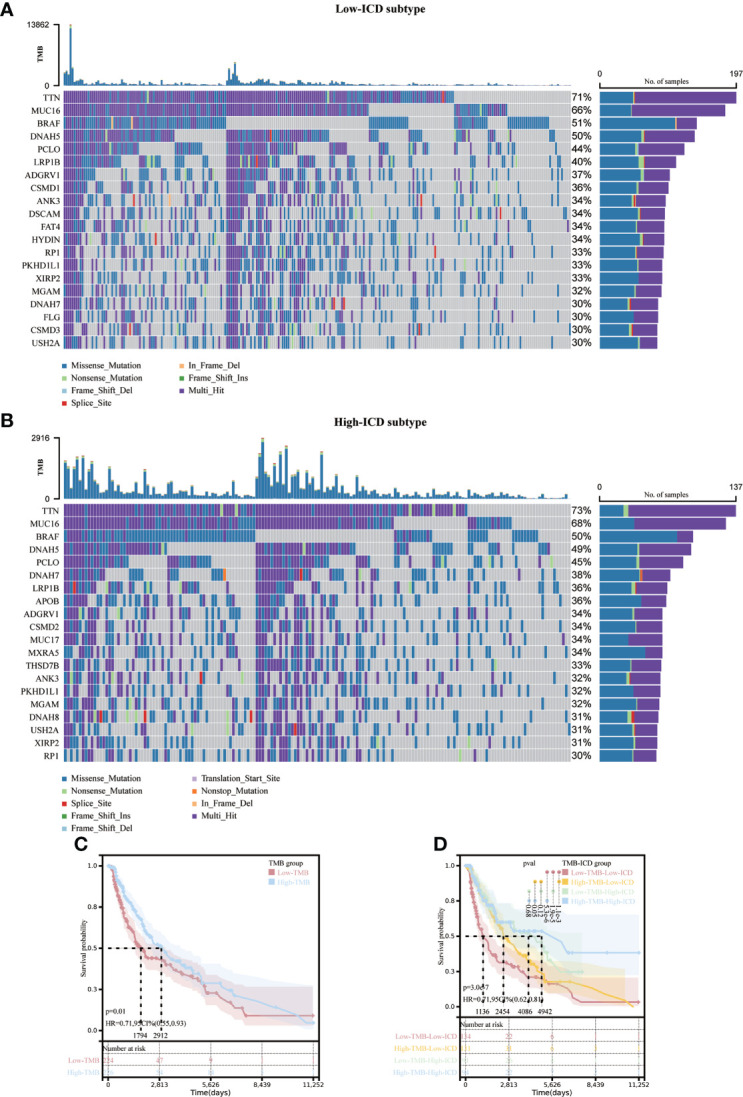
Correlation of different ICD subtypes with somatic mutations. Oncoprint visualizes the ten most frequently mutated genes in the ICD-low subtype **(A)** and the ICD-high subtype **(B)**. Kaplan-Meier curves for the high TMB and low TMB groups **(C)**.Kaplan-Meier curve stratification of patients according to TMB and ICD genetic characteristics **(D)**.

There were different somatic mutation profiles in the high and low ICD subtypes in SKCM. TTN, MUC16, BRAF and DNAH5 had the highest mutation frequencies. They were 71%, 66%, 51%, 50% in the low ICD subtype and 73%, 68%, 50%, 49% in the high ICD subtype. the high ICD subtype tumor mutation burden (TMB) was slightly higher. (**Figures 4A, B**). To reveal genetic variation in ICD subtypes, we plotted K-M survival curves based on TMB levels, indicating longer OS in the high TMB group (*p* = 0.01, **Figure 4C**). We divided the patients into four subgroups based on the median ICD subtype and TMB, as shown in **Figure 4D**, with the longest OS in the high TMB value and high ICD subgroups, and the shortest OS in the low TMB value and low ICD subgroups (*p* < 0.001).

**Figure 5:**
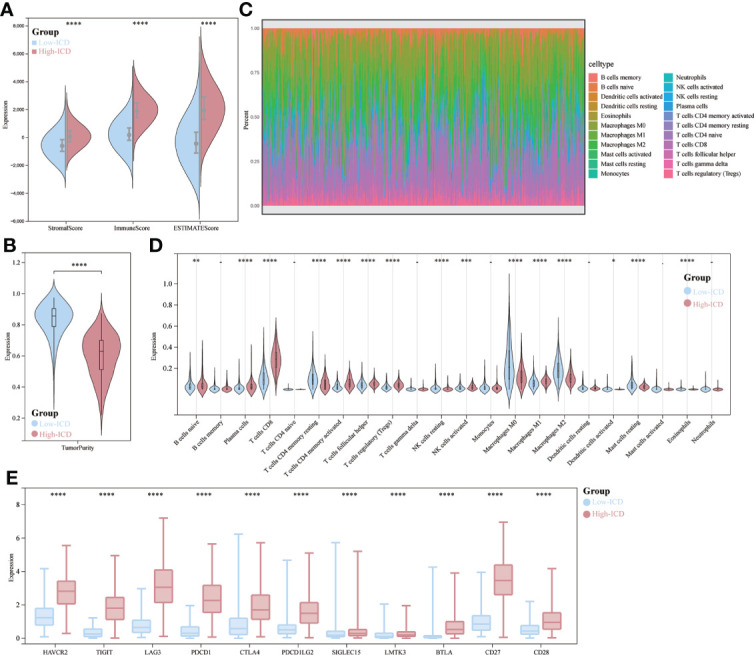
Immune landscape of ICD-high and ICD-low subtypes. **(A, B)** Fractional violin plots showing median and interquartile estimates of each immune score and tumor purity score; **(C)** relative proportions of immune infiltration in ICD-high and ICD-low subtypes; **(D)** Violin plots showing immune cells that differ significantly between subtypes; **(E)** box plots indicating differential expression of multiple immune checkpoints. *p < 0.05, **p < 0.01, ***p < 0.001, & ****p < 0.0001.

There is growing evidence that ICDs strongly influence the activation of specific antitumor immune responses. In the present study, we analyzed the composition of the tumor microenvironment between the two subtypes. Overall, the high ICD subtype had higher immune scores and lower tumor purity than the low ICD subtype (**Figures 5A, B**). **Figure 5C** demonstrates the percentage of immune infiltrating cells in each sample from SKCM patients. Next, we assessed the correlation between gene expression and infiltration of 22 immune cells by collecting 467 samples from TCGA using the CIBERSORT method in combination with the LM22 marker matrix. We found significant differences in naive B cells, plasma cells, CD8 T cells, resting CD4 T memory cells, activated CD4 T cells, helper T cells, regulatory T cells, resting NK cells, activated NK cells, macrophages M0, M1, M2 activated myeloid dendritic cells and eosinophils. Of these, CD8 T cells, B-cell plasma, activated CD4 T memory cells, helper T cells, and regulatory T cells were significantly upregulated, whereas they were significantly downregulated in macrophages M0 and M2 (**Figure 5D**). In addition, ICD high subtypes were upregulated in 12 common immune checkpoints, and the opposite was true for ICD low subtypes (**Figure 5E**). These results suggest that these ICD-related genes are more closely associated with immunotherapy.

### Construction and verification of ICD risk signatures

**Figure 6:**
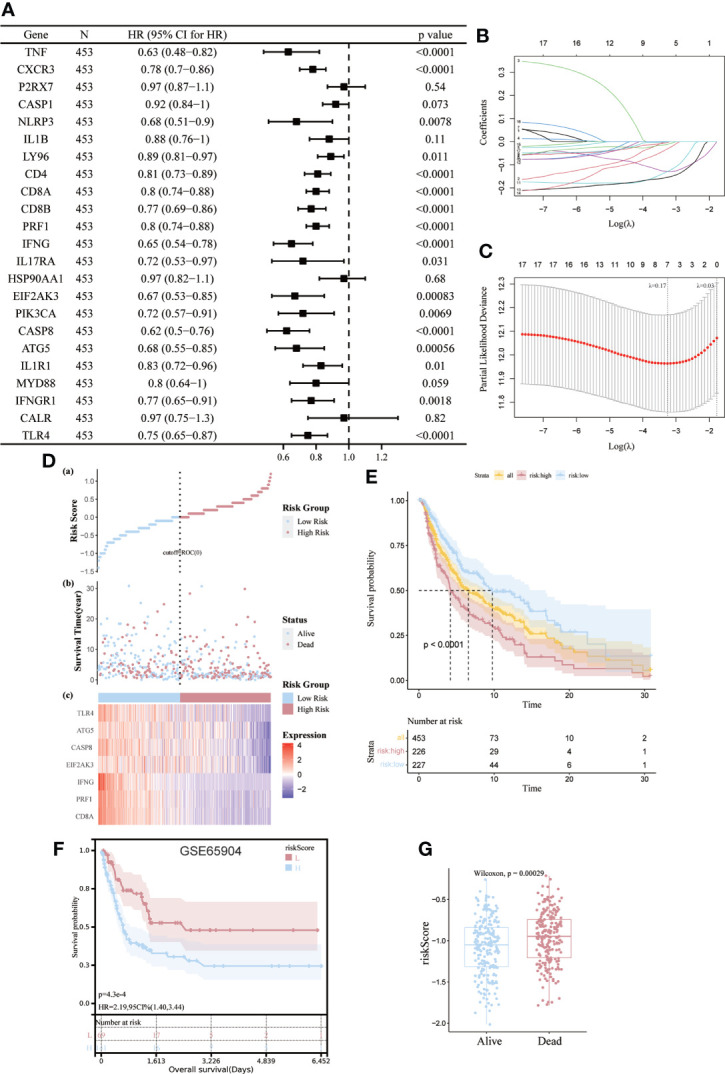
Construction and validation of risk models. **(A)** Univariate Cox analysis to assess the prognostic value of ICD genes in terms of OS; **(B, C)** Lasso Cox analysis to identify the seven genes most associated with OS in the TCGA dataset; **(D)** Heat map of the risk score distribution, survival status and prognostic 7-gene signature for each patient in the TCGA database; **(E, F)** Kaplan- Meier analysis demonstrating the prognostic significance of the risk model in the TCGA and GSE65904 cohort; **(G)** Box plots representing the relationship between patient survival status and risk score.

We then developed a prognostic model predicated on ICD-related genes. 17 ICD-related genes were found to be significantly associated with patient OS by Cox univariate analysis (**Figure 6A**). Seven differential genes (CD8A, PRF1, IFNG, EIF2AK3, CASP8, ATG5, TLR4) were detected and selected as predictive models in LASSO regression analysis We determined the optimal penalty parameter lambda and calculated the corresponding coefficient criterion based on the minimum criterion through 1,000-fold cross-validation. (**Figures 6B, C**). Therefore, a risk prediction model for seven ICD-related genes was constructed. riskScore=(-0.129496376834374)*CD8A+(-0.0034750461455518)*PRF1+(-0.0113364597991046)*IFNG+(-0.129296018992947)*EIF2AK3+(- 0.154479588206008)*CASP8+(-0.0312155447535074)*ATG5+(-0.00138961924731843)*TLR4. Calculate the risk score for each patient. Based on the median risk score, 453 SKCM patients were divided into high-risk group (n=226) and low-risk group (n=227). Low-risk group with low risk score and vice versa. Risk score distribution and survival status show more surviving states in the low-risk cohort than in the high-risk cohort (**Figure 6D**).

The importance of this risk profile in SKCM was further determined using KM analysis. The TCGA cohort found a significant difference between high-risk scores corresponding to poorer OS, which was further corroborated by comparable results in the GEO cohort (**Figures 6E, F**). Mortality was significantly higher (p=0.00029) for patients in the high-risk group compared to the low-risk group by Wilcoxon statistics (**Figure 6G**).

### The Association of ICD risk signature with tumor microenvironment

**Figure 7:**
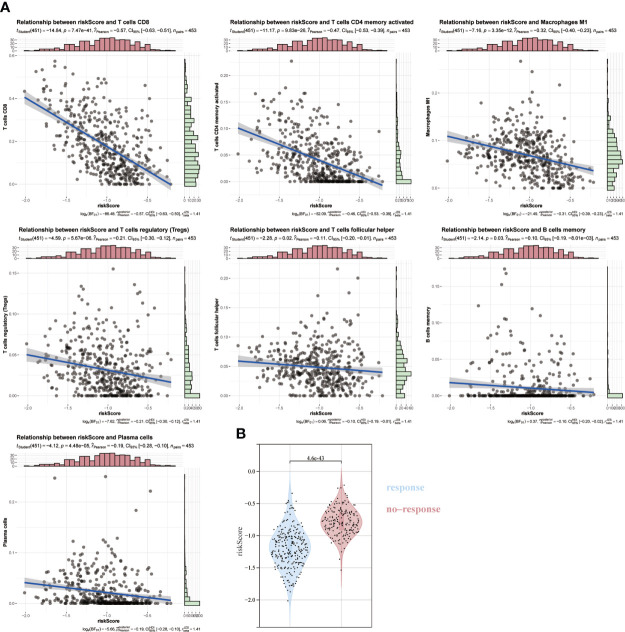
Association of ICD risk score with the tumor microenvironment. **(A)** Scatter plot showing the correlation of risk score with infiltration of CD8, activated NK cells and activated CD4 memory cells. **(B)** Plot showing the association of ICD risk score with response to immunotherapy.

Given the essential biological role of ICD in the anti-tumor immune response, we confirmed the association between ICD risk scores and the tumor microenvironment in 467melanoma patients by Spearman correlation analysis. We verified that patients with higher risk scores were negatively associated with memory B cells, CD8, Tregs cells, helper T cells, activated CD4 memory cells, and plasma cells (**Figure 7A**). We then used TIDE to assess the predictive value of ICD risk characteristics for the potential clinical efficacy of immunotherapy. The results showed that the ICD risk score was lower in the immunotherapy-responsive group, implying that patients with a low ICD risk score are more suitable for immunotherapy (**Figure 7B**).

### The independent prognostic value of ICD risk characteristics was assessed using univariate and multifactorial Cox analyses

**Figure 8:**
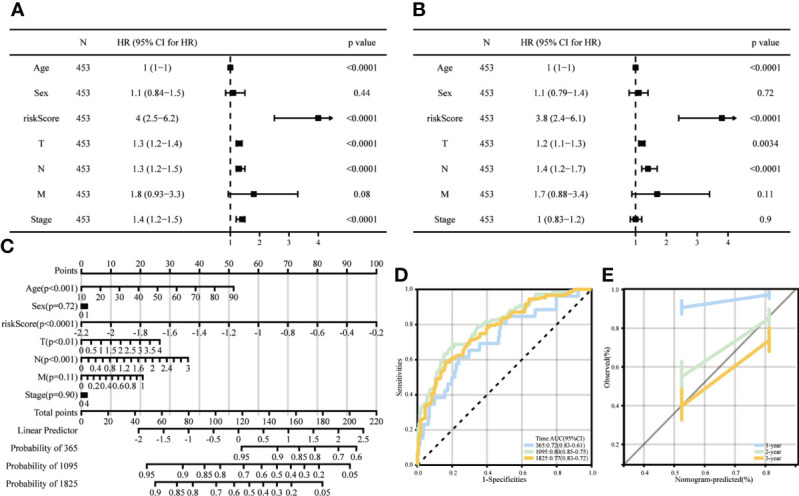
Columnar line graphs of TME risk characteristics for predicting survival in melanoma patients. **(A, B)** Forest plots from univariate and multivariate Cox regression analyses, including risk score, age, sex, grade and stage. **(C)** Columnar plots of risk scores and stages were used to predict survival at 1, 3 and 5 years in melanoma patients. **(D)** Time-dependent ROC curve analysis shows that column line graphs are reliable and stable predictors of OS at one year, three years and five years. **(E)** Calibration curves show the column line graphs’ predicted (x-axis) and actual survival probabilities (y-axis).

Univariate analysis showed that a high ICD risk score and stage were significantly associated with poorer OS (**Figure 8A**). Multi-factor analysis showed that the ICD risk score could be an independent prognostic factor for patients with SKCM (**Figure 8B**). We then constructed nomogram based on risk score and stage to examine the probability of survival for 1, 3 and 5-year survivors (**Figure 8C**). The area values for 1-year, 3-year and 5-year survival under the ROC curve were 0.72, 0.8 and 0.77, respectively, indicating accurate discrimination (**Figure 8D**). Also, calibration curves were plotted to assess the agreement between the predicted and actual values of OS (**Figure 8E**).

## Discussion

Many preclinical studies have identified the ICD as an important predictor of effective anti-tumor immunity (11, 27). However, it has not yet been fully established as a method for discovering prognostic biomarkers. Although we know that higher immune cell infiltration is associated with better treatment outcomes. But much emerging evidence suggests that prognostic analysis needs to take into account immune tumour heterogeneity. abhishek et al. (26) also made some conflicting observations about the prognostic impact of ICD-related risk signals. For example, identified immunostimulatory factors (e.g. IFNG in lung cancer) are associated with a negative prognosis, whereas immunosuppressive factors (e.g. FOXP3/IL10 in ovarian cancer) are associated with a positive prognosis. Many of the different cancer types do not show the expected outcomes with their immunotherapy. Immunogenic death has not been studied in depth in melanoma. In this study, we clustered all samples into high and low ICD subtypes based on ICD-related genes in 467 cases. The high ICD subgroup is associated with a better prognosis and higher levels of immune cell infiltration than the low ICD subgroup. Some studies have reported better efficacy of tumor-applied immunosuppression when suppressor receptors are highly expressed (28), which is consistent with our findings. Among the different subtypes of ICD, the frequency of mutations was slightly higher in the high ICD subtype than in the low subtype group. Previously published studies have shown that tumor mutation burden (TMB) is strongly associated with response to ICIS therapy (29, 30).TMB reflects the presence of somatic mutant loci in the tumor genome that contribute to the generation of new antigens and immunogenicity, leading to T cell responses (31). CD8 T cell levels in SKCM are positively correlated with neoantigen load, and tumor types with high mutational load may have better immunotherapeutic outcomes. However, tumor specificity makes a difference in outcomes (32), and mutational load and immunotherapeutic response need further investigation.

The ICD-related genes analyzed were identified by Abhishek et al. (26) through the Web of Knowledge, Scopeus and PubMed collections and were studied *in vitro* and *in vivo* experiments. In our analysis, a prognostic risk profile was constructed for these seven ICD-related genes strongly associated with the prognosis of SKCM patients, classifying SKCM patients into high- and low-risk cohorts. For OS, this risk profile showed a high predictive value and may serve as an independent prognostic indicator for SKCM patients. At the same time, we elucidated by TIDE analysis that risk scores were higher in immunotherapy non-responders, reinforcing that people with high ICD subtypes have a better response to immunotherapy.

It has been noted that the oncogenic effects of model genes and the efficacy of anti-tumor therapy are closely related. CD8A and EIF2AK3 are essential ICD genes in prognostic models. It has been reported that in triple-negative breast cancer (TNBC) patients, the inflammatory DAMP subtype has the most significant proportion of CD8 T cells and is associated with the best predictive outcome, that M2-like macrophages in the suppressor subtype of DAMPs significantly increase inflammation, and that triple-negative breast cancers with high CD8A expression benefit from ICI (33, 34). Xu et al. (33) study also showed that ICD was significantly enhanced by upregulating CD8A and releasing HMGB1 in tumor tissues, which resulted in an enhanced role of immune cells infiltrating the tumor microenvironment after cancer antigen exposure in the presence of ICD (35, 36). In contrast, EIF2A3 is eukaryotic translation initiation factor 2α kinase three. It was demonstrated that eIF2α phosphorylation constitutes a convenient biomarker to predict cancer cell immunogenicity in medically induced stress (37). When we classified SKCM patients into high and low risk based on seven genes, including CD8A and EIF2AK3, the high-risk group was associated with a poor prognosis, and the high-risk score could be for as an independent prognostic indicator for SKCM. This suggests an essential value in predicting the prognosis of SKCM patients.

In conclusion, we have further demonstrated the close correlation between the high ICD subgroup and the immune microenvironment through the grouping of high and low ICD and the risk profile consisting of CD8A, PRF1, IFNG, EIF2AK3, CASP8, ATG5, TLR4 to further identify relevant biomarkers of ICD in SKCM, bringing more accurate utility for immunotherapy of SKCM.

## Data availability statement

The datasets presented in this study can be found in online repositories. The names of the repository/repositories and accession number(s) can be found in the article/Supplementary Material.

## Author contributions

XF worked on the design of the research idea, revised the manuscript and finalized it. YH analyzed the data and wrote the manuscript. QC optimized the research methods and combined images. XX searched the data and assisted in writing the manuscript. SG collated the cited literature and helped to enrich the article. All authors contributed to the article and approved the submitted version.

## Funding

This study was supported by The Regional Science and Technology Support Project (2022E02048).

## Acknowledgments

We would like to acknowledge the TCGA, GTEx and the GEO (GSE140082) network for providing data.

## Conflict of interest

The authors declare that the research was conducted in the absence of any commercial or financial relationships that could be construed as a potential conflict of interest.

## Publisher’s note

All claims expressed in this article are solely those of the authors and do not necessarily represent those of their affiliated organizations, or those of the publisher, the editors and the reviewers. Any product that may be evaluated in this article, or claim that may be made by its manufacturer, is not guaranteed or endorsed by the publisher.

## References

[1] Rebecca VW Somasundaram R Herlyn M. Pre-clinical modeling of cutaneous melanoma. Nat Commun (2020) 11(1):2858. doi: 10.1038/s41467-020-15546-9 32504051 PMC7275051

[2] Bray F Ferlay J Soerjomataram I Siegel RL Torre LA Jemal A. Global cancer statistics 2018: GLOBOCAN estimates of incidence and mortality worldwide for 36 cancers in 185 countries. CA Cancer J Clin (2018) 68(6):394–424. doi: 10.3322/caac.21492 30207593

[3] Bittner M Meltzer P Chen Y Jiang Y Seftor E Hendrix M. Molecular classification of cutaneous malignant melanoma by gene expression profiling [J]. Nature (2000) 406(6795):536–40. doi: 10.1038/35020115 10952317

[4] Bagchi S Yuan R Engleman EG. Immune checkpoint inhibitors for the treatment of cancer: Clinical impact and mechanisms of response and resistance. Annu Rev Pathol (2021) 16:223–49. doi: 10.1146/annurev-pathol-042020-042741 33197221

[5] Carlino MS Larkin J Long GV. Immune checkpoint inhibitors in melanoma. Lancet (2021) 398(10304):1002–14. doi: 10.1016/S0140-6736(21)01206-X 34509219

[6] Wolchok JD Chiarion-Sileni V Gonzalez R Rutkowski P Grob J-J Cowey CL. Overall survival with combined nivolumab and ipilimumab in advanced melanoma. N Engl J Med (2017) 377(14):1345–56. doi: 10.1056/NEJMoa1709684 PMC5706778 28889792

[7] Schadendorf D Vaubel J Livingstone E Zimmer L. Advances and perspectives in immunotherapy of melanoma. Ann Oncol (2012) 23 Suppl 10:x104–8. doi: 10.1093/annonc/mds321 22987943

[8] Robert C Schachter J Long GV Arance A Grob JJ Mortier L. Pembrolizumab versus ipilimumab in advanced melanoma. N Engl J Med (2015) 372(26):2521–32. doi: 10.1056/NEJMoa1503093 25891173

[9] Tang YZ Szabados B Leung C Sahdev A. Adverse effects and radiological manifestations of new immunotherapy agents. Br J Radiol (2019) 92(1093):20180164. doi: 10.1259/bjr.20180164 30281331 PMC6435070

[10] Li Z Lai X Fu S Ren L Cai H Zhang H. Immunogenic cell death activates the tumor immune microenvironment to boost the immunotherapy efficiency. Adv Sci (Weinh) (2022) 9:e2201734. doi: 10.1002/advs.202201734 35652198 PMC9353475

[11] Krysko DV Garg AD Kaczmarek A Krysko O Agostinis P Vandenabeele P. Immunogenic cell death and DAMPs in cancer therapy. Nat Rev Cancer (2012) 12(12):860–75. doi: 10.1038/nrc3380 23151605

[12] Birmpilis AI Paschalis A Mourkakis A Christodoulou P Kostopoulos IV Antimissari E. Immunogenic cell death, DAMPs and prothymosin α as a putative anticancer immune response biomarker. Cells (2022) 11(9):1415. doi: 10.3390/cells11091415 35563721 PMC9102069

[13] Xiao X Liang S Zhao Y Pang M Ma P Cheng Z. Multifunctional carbon monoxide nanogenerator as immunogenic cell death drugs with enhanced antitumor immunity and antimetastatic effect. Biomaterials (2021) 277:121120. doi: 10.1016/j.biomaterials.2021.121120 34508956

[14] Bao X Xie L. Targeting purinergic pathway to enhance radiotherapy-induced immunogenic cancer cell death. J Exp Clin Cancer Res (2022) 41(1):222. doi: 10.1186/s13046-022-02430-1 35836249 PMC9284706

[15] Li Y Song Z Han Q Zhao H Pan Z Lei Z. Targeted inhibition of STAT3 induces immunogenic cell death of hepatocellular carcinoma cells via glycolysis. Mol Oncol (2022) 16:2861–80. doi: 10.1002/1878-0261.13263 PMC9348600 35665592

[16] Qi J Jin F Xu X Du Y. Combination cancer immunotherapy of nanoparticle-based immunogenic cell death inducers and immune checkpoint inhibitors. Int J Nanomedicine (2021) 16:1435–56. doi: 10.2147/IJN.S285999 PMC7910111 33654395

[17] Wilkerson MD Hayes DN. ConsensusClusterPlus: a class discovery tool with confidence assessments and item tracking. Bioinformatics (2010) 26(12):1572–3. doi: 10.1093/bioinformatics/btq170 PMC2881355 20427518

[18] Phipson B Lee S Majewski IJ Alexander WS Smyth GK. Robust hyperparameter estimation protects against hypervariable genes and improves power to detect differential expression. Ann Appl Stat (2016) 10(2):946–63. doi: 10.1214/16-AOAS920 PMC5373812 28367255

[19] Wu T Hu E Xu S Chen M Guo P Dai Z. clusterProfiler 4.0: A universal enrichment tool for interpreting omics data. Innovation (N Y) (2021) 2(3):100141. doi: 10.1016/j.xinn.2021.100141 PMC8454663 34557778

[20] Becht E Giraldo NA Lacroix L Buttard B Elarouci N Petitprez F. Estimating the population abundance of tissue-infiltrating immune and stromal cell populations using gene expression. Genome Biol (2016) 17(1):218. doi: 10.1186/s13059-016-1070-5 27765066 PMC5073889

[21] Newman AM Liu CL Green MR Gentles AJ Feng W Xu Y. Robust enumeration of cell subsets from tissue expression profiles. Nat Methods (2015) 12(5):453–7. doi: 10.1038/nmeth.3337 PMC4739640 25822800

[22] Fu J Li K Zhang W Wan C Zhang J Jiang P. Large-Scale public data reuse to model immunotherapy response and resistance. Genome Med (2020) 12(1):21. doi: 10.1186/s13073-020-0721-z 32102694 PMC7045518

[23] Mayakonda A Lin D-C Assenov Y Plass C Koeffler HP. Maftools: efficient and comprehensive analysis of somatic variants in cancer [J]. Genome Res (2018) 28(11):1747–56. doi: 10.1101/gr.239244.118 PMC6211645 30341162

[24] Friedman J Hastie T Tibshirani R. Regularization paths for generalized linear models *via* coordinate descent. J Stat Softw (2010) 33(1):1–22. 20808728 PMC2929880

[25] Therneau T Therneau T Grambsch P. Modeling survival data: Extending the cox model (statistics for biology and health). (2000).

[26] Garg AD De Ruysscher D Agostinis P. Immunological metagene signatures derived from immunogenic cancer cell death associate with improved survival of patients with lung, breast or ovarian malignancies: A large-scale meta-analysis. Oncoimmunology (2016) 5(2):e1069938. doi: 10.1080/2162402X.2015.1069938 27057433 PMC4801472

[27] Kroemer G Galluzzi L Kepp O Zitvogel L. Immunogenic cell death in cancer therapy. Annu Rev Immunol (2013) 31:51–72. doi: 10.1146/annurev-immunol-032712-100008 23157435

[28] Chiang N-J Hou Y-C Tan KT Tsai H-W Lin Y-J Yeh Y-C. The immune microenvironment features and response to immunotherapy in EBV-associated lymphoepithelioma-like cholangiocarcinoma. Hepatol Int (2022) 16:1137–49. doi: 10.1007/s12072-022-10346-3 35780451

[29] Ballhausen A Przybilla MJ Jendrusch M Haupt S Pfaffendorf E Seidler F. The shared frameshift mutation landscape of microsatellite-unstable cancers suggests immunoediting during tumor evolution. Nat Commun (2020) 11(1):4740. doi: 10.1038/s41467-020-18514-5 32958755 PMC7506541

[30] Georgiadis A Durham JN Keefer LA Bartlett BR Zielonka M Murphy D. Noninvasive detection of microsatellite instability and high tumor mutation burden in cancer patients treated with PD-1 blockade. Clin Cancer Res (2019) 25(23):7024–34. doi: 10.1158/1078-0432.CCR-19-1372 PMC6892397 31506389

[31] Reuben A Zhang J Chiou S-H Gittelman RM Li J Lee W-C. Comprehensive T cell repertoire characterization of non-small cell lung cancer. Nat Commun (2020) 11(1):603. doi: 10.1038/s41467-019-14273-0 32001676 PMC6992630

[32] Mcgrail DJ Pilié PG Rashid NU Voorwerk L Slagter M Kok M. High tumor mutation burden fails to predict immune checkpoint blockade response across all cancer types. Ann Oncol (2021) 32(5):661–72. doi: 10.1016/j.annonc.2021.02.006 PMC8053682 33736924

[33] Xu M Lu J-H Zhong Y-Z Jiang J Shen Y-Z Su J-Y. Immunogenic cell death-relevant damage-associated molecular patterns and sensing receptors in triple-negative breast cancer molecular subtypes and implications for immunotherapy. Front Oncol (2022) 12:870914. doi: 10.3389/fonc.2022.870914 35444934 PMC9013947

[34] Katsuta E Yan L Opyrchal M Kalinski P Takabe K. Cytotoxic T-lymphocyte infiltration and chemokine predict long-term patient survival independently of tumor mutational burden in triple-negative breast cancer. Ther Adv Med Oncol (2021) 13:17588359211006680. doi: 10.1177/17588359211006680 33868461 PMC8024454

[35] Serrano Del Valle A Anel A Naval J Marzo I. Response: Commentary: Immunogenic cell death and immunotherapy of multiple myeloma. Front Cell Dev Biol (2019) 7:306. doi: 10.3389/fcell.2019.00306 31828071 PMC6890598

[36] Sansone C Bruno A Piscitelli C Baci D Fontana A Brunet C. Natural compounds of marine origin as inducers of immunogenic cell death (ICD): Potential role for cancer interception and therapy. Cells (2021) 10:231. doi: 10.3390/cells10020231. 33504012 PMC7912082

[37] Bezu L Wu Chuang A Humeau J Kroemer G Kepp O. Quantification of eIF2alpha phosphorylation during immunogenic cell death. Methods Enzymol (2019) 629:53–69. doi: 10.1016/bs.mie.2019.04.010 31727256

